# Silent Verb Generation During fMRI Reveals Post-Radiotherapy Alterations in Cerebellar–Cerebral Language Regions in Patients Treated for Medulloblastoma

**DOI:** 10.3390/cancers18172795

**Published:** 2026-08-28

**Authors:** Josue Luiz Dalboni da Rocha, Ping Zou Stinnett, Stu McAfee, Carolina Torres-Rojas, Matthew A. Scoggins, Askery Canabarro, Pankaj Pandey, Thomas E. Merchant, Giles Robinson, Amar Gajjar, Heather M. Conklin, Ranganatha Sitaram

**Affiliations:** 1 Department of Radiology, St. Jude Children’s Research Hospital, Memphis, TN 38105, USA; 2 Department of Psychology and Biobehavioral Sciences, St. Jude Children’s Research Hospital, Memphis, TN 38105, USA; 3 Quantum Research Center, Technology Innovation Institute, Abu Dhabi P.O. Box 9639, United Arab Emirates; 4 Núcleo de Ciências Exatas (NCEx), Campus Arapiraca, Federal University of Alagoas, Arapiraca 57309-005, AL, Brazil; 5 Department of Radiation Oncology, St. Jude Children’s Research Hospital, Memphis, TN 38105, USA; 6 Department of Oncology, St. Jude Children’s Research Hospital, Memphis, TN 38105, USA; 7 Department of Pediatric Medicine, St. Jude Children’s Research Hospital, Memphis, TN 38105, USA

**Keywords:** medulloblastoma, radiotherapy, fMRI, cerebellum, neurocognition

## Abstract

**Background/Objectives:** Medulloblastoma is the most common malignant pediatric brain tumor. Although radiotherapy improves survival, it is often associated with neurocognitive impairments affecting language and executive functions in developing brains. We investigated treatment-related changes in task-evoked activation using silent verb-generation functional magnetic resonance imaging (fMRI). **Methods:** Thirty-one children and adolescents with medulloblastoma enrolled on SJMB12 (18 males; mean age 14.1 ± 4.7 years) completed visual and auditory noun-cued covert verb generation during fMRI before radiotherapy and again within 6 weeks after completing radiotherapy. A two-stage, nested feature-selection framework coupled with a linear Support Vector Machine differentiated pre- from post-irradiation scans using fully nested leave-one-subject-out cross-validation, in which the sign-consistency threshold was re-selected within each training fold, and was evaluated against the 50% chance level of the paired forced-choice design. **Results:** In fully nested leave-one-subject-out evaluation, the pipeline correctly ordered the paired pre- and post-radiotherapy scans for 22 of 31 participants (71.0%; 95% CI, 52.0–85.8%; one-sided permutation *p* = 0.015). Descriptive cohort-derived mapping at the modal inner-loop sign-consistency threshold implicated reduced activity in bilateral cerebellar hemispheres (Crus I/II, lobules VI–VIII), left inferior frontal gyrus, left insula, left putamen, supragenual anterior cingulate, and left middle temporal gyrus. Reduced task-negative responses were observed in a large cluster overlapping right precuneus, right superior parietal, right paracentral, and right postcentral cortices, alongside increased engagement of the left supramarginal gyrus and left inferior parietal lobule. **Conclusions:** In this single-center pilot cohort, silent verb-generation fMRI detected early post-radiotherapy activation changes within cerebellar–cerebral language and control networks. These findings require independent validation, test–retest controls, and longitudinal neurocognitive outcomes before being interpreted as clinical biomarkers.

## Simple Summary

Children treated for medulloblastoma often survive their disease but remain at risk for later difficulties with language, attention, and working memory. This study used functional MRI during a silent verb-generation task to examine how brain activity changed soon after radiotherapy in 31 patients. An internally cross-validated machine-learning analysis distinguished pre- from early post-radiotherapy scans above chance in this single-center cohort, and the discriminative pattern involved cerebellar, frontal, temporal, cingulate, and parietal regions that support language production and cognitive control. Because this study did not include an independent validation cohort, a non-irradiated test–retest control group, or concurrent neurocognitive outcomes, these imaging findings should not be interpreted as validated biomarkers or clinical predictors. Rather, they indicate that longitudinal task fMRI is feasible for detecting early treatment-associated network changes that warrant prospective validation in larger cohorts with behavioral outcomes and harmonized acquisition.

## 1. Introduction

Medulloblastoma arises in the posterior fossa and remains the most common malignant pediatric brain tumor, accounting for approximately 20% of all childhood brain malignancies [1]. Advances in adjuvant therapy, especially risk-adapted radiotherapy have significantly increased survival rates [2]. However, patients often face a range of life-altering effects, with neurocognitive deficits among the most pronounced and debilitating [3]. These deficits affect domains such as language, working memory, processing speed, and executive function, ultimately impacting academic achievement and quality of life [4]. Literature indicates that the developing brain is particularly vulnerable to ionizing radiation, which disrupts proliferative zones and myelination in white matter tracts crucial for language and cognitive functions [5].

Task-based functional magnetic resonance imaging (fMRI) based on blood oxygen level-dependent (BOLD) contrast provides a noninvasive method for measuring task-related changes in local hemodynamic responses coupled to neural activity [6,7]. In pediatric neuro-oncology, task fMRI has been used to characterize alterations in language, reading, attention, and executive networks after treatment, including reading-related activation changes in medulloblastoma survivors [8]. Language fMRI paradigms such as silent verb generation and orthographic lexical retrieval typically activate frontal and temporal language regions, as well as the cerebellum [9,10,11].

Prior fMRI studies in pediatric brain tumor and medulloblastoma populations have shown that survivorship can be accompanied by altered recruitment of distributed frontal, temporal, parietal, and cerebellar regions during language, reading, working-memory, and executive tasks [8,12,13]. These studies support the biological plausibility of treatment-related functional reorganization, but most have been cross-sectional, have focused on long-term survivors, or have not isolated the early post-radiotherapy window. Therefore, what remains unclear is whether task-evoked BOLD changes are already detectable within weeks of radiotherapy completion, before chemotherapy exposure, and whether such changes can be summarized at the individual patient level using internally cross-validated multivariate pattern analysis. The novelty of the present study is thus the paired TP2-TP1 design in medulloblastoma and the exploratory identification of an early treatment-associated cerebellar–cerebral language network activation signature.

The cerebellum is increasingly recognized for its role in higher cognitive functions, including language planning and executive processing [14,15]. Mounting evidence shows that cerebellar dysfunction can lead to impairment in linguistic, executive, and affective domains [16,17]. Given these insights, the cerebellum is a pivotal region of interest in understanding the neural underpinnings of post-radiation deficits in medulloblastoma patients.

The present study had four objectives with different levels of inference. First, we characterized the distribution of task-related activation during silent verb generation before radiotherapy and within six weeks after radiotherapy. Second, we quantified within-subject regional changes in task-evoked BOLD activation from TP1 to TP2. Third, as a pilot feasibility analysis, we tested whether an internally cross-validated multivariate classifier could distinguish paired pre- from early post-radiotherapy scans above chance. Fourth, we summarized the cohort-derived multivariate pattern using descriptive patient-level expression metrics in percent-signal-change units. The primary novelty is the paired early post-radiotherapy design and the multivariate characterization of treatment-associated activation changes in pediatric medulloblastoma. We hypothesized, from TP1 to TP2, silent verb-generation fMRI would show within-subject changes in task-evoked activation within cerebellar–cerebral language and control regions. We further hypothesized that these paired activation changes would be detectable above chance by an internally cross-validated multivariate classifier.

## 2. Materials and Methods

### 2.1. Participants

Thirty-one pediatric medulloblastoma patients (18 males; mean age at irradiation: 14.1 ± 4.7 years) were prospectively enrolled on the St. Jude SJMB12 clinical trial (ClinicalTrials.gov identifier: NCT01878617) at a tertiary pediatric oncology center. Participant demographic and clinical characteristics are summarized in Table 1. Inclusion criteria for fMRI were:

(1) Histologically confirmed medulloblastoma.
(2) Age greater than or equal to 6 years and less than 22 years at the time of diagnosis.
(3) No previous radiotherapy, chemotherapy, or other brain tumor-directed therapy other than corticosteroid therapy and surgery.
(4) Patients were required to begin treatment as outlined in the protocol within 36 days of definitive surgery (day of surgery is day 0; definitive surgery includes second surgeries to resect residual tumor).
(5) Females must not be pregnant or breast-feeding. Female participants >10 years of age or post-menarche must have a negative serum or urine pregnancy test prior to enrollment.
(6) Planned standardized post-operative radiotherapy regimen following maximal safe resection.
(7) Ability to undergo an MRI session without sedation or with minimal sedation.
(8) No contraindications to MRI.

The eight participants recorded as having a history of posterior fossa syndrome are the same individuals listed under “Complete mutism” (n = 3) and “Reduced speech” (n = 5); these categories are not additive. Patients with diagnoses other than medulloblastoma or severe neurologic deficits precluding task participation (e.g., significant aphasia, profound sensorimotor impairment) were excluded. Written informed consent and assent procedures were completed as described in the Informed Consent Statement. Additional ethical approval details are provided in the Institutional Review Board Statement.

The post-operative neurologic categories in Table 1 refer to the immediate post-operative clinical course and should not be interpreted as task performance at the fMRI visit. Inclusion in the analyzed fMRI cohort required sufficient alertness, comprehension of the noun cues, and ability to attempt covert verb generation at the time of scanning. Patients with post-operative mutism or reduced speech were included only if they had recovered sufficiently by TP1 to understand instructions and attempt the silent task. Participants unable to understand or attempt the task at TP1 were excluded from the fMRI analysis.

### 2.2. Study Design

All 31 participants underwent fMRI at TP1 and TP2. The primary comparison was TP1 versus TP2: TP1 was acquired at a median of 28 days after definitive surgery (range, 13–44 days) and within 1 week before the start of radiotherapy. Individual surgery-to-TP1 intervals are provided in Supplementary Table S1. TP2 was acquired within 6 weeks after radiotherapy and before initiation of adjuvant chemotherapy. Chemotherapy was initiated after TP2 and before TP3. Thus, the TP2–TP1 contrast was designed to exclude chemotherapy-related confounding and to capture the early post-radiotherapy interval, while recognizing that post-operative recovery, edema resolution, task repetition, and time since surgery may also contribute to the observed BOLD changes.

Although 31 participants had paired TP2–TP1 fMRI and were included in the primary analysis, only 8 participants had complete usable silent verb-generation fMRI data at all the five protocol-defined visits. Later timepoints were incomplete because of missed protocol visits or fMRI acquisition. Because the later protocol-defined visits were available only for these 8 participants, any model fitted across TP1–TP5 would rest on the same subset at TP3–TP5 regardless of the estimation approach, including a mixed model fitted to all available trajectories, and a model-based fit would express that dependence implicitly rather than explicitly. We therefore restricted the longitudinal TP1–TP5 analysis to complete cases and treat it as exploratory and hypothesis-generating. The primary inference remains the paired TP2–TP1 contrast available for the full cohort. The later protocol-defined visits were labeled chronologically as TP3 (post-chemotherapy protocol follow-up), TP4 (three-month follow-up after TP3), and TP5 (six-month follow-up after TP4).

### 2.3. fMRI Task: Silent Verb Generation

Participants completed a silent verb-generation task during two separate fMRI runs: one with visually presented nouns and the other with auditory presentation. Each session followed a block design alternating between stimulation periods (for verb generation) and fixation periods (for rest). During stimulation blocks, participants were instructed to silently generate a verb associated with each presented noun (e.g., hearing or seeing “car” and thinking “drive”). Each stimulation block lasted 20 s. In the visual modality, nouns were shown on the screen every 3 s for 1 s. In the auditory modality, each noun was spoken once during the stimulation period, following the same timing structure. Resting blocks also lasted 20 s and displayed a fixation cross continuously.

Because the verb-generation paradigm was intentionally covert, trial-level accuracy could not be recorded. To support compliance, participants received standardized task instructions and practiced example noun-verb associations before scanning until they could explain or demonstrate the task. During scanning, the MRI team monitored wakefulness.

### 2.4. Image Acquisition

All fMRI data included in the present analysis were acquired on a single Siemens Skyra 3T MRI scanner (Siemens Healthineers, Erlangen, Germany). High-resolution T1-weighted structural images (voxel size = 0.5 × 0.5 × 1 mm^3^) were obtained. Functional data were collected using a gradient-echo echo-planar imaging sequence sensitive to BOLD contrast (TR = 2000 ms, voxel size = 3 × 3 × 4 mm^3^, matrix size = 64 × 64 × 36). Slices covered the whole brain, including the cerebellum.

### 2.5. Image Preprocessing

Functional image preprocessing was performed with the SPM software Version 12 (http://www.fil.ion.ucl.ac.uk/spm/, accessed on 27 March 2025) [18]. Steps included:

1. Realignment: Motion correction by aligning all functional volumes to a mean reference volume. Head motion was quantified from the six SPM realignment parameters for each fMRI run. Framewise displacement was calculated as the sum of the absolute derivatives of the three translational parameters and the three rotational parameters, with rotations converted to millimeters using a 50 mm radius. For each scan, we summarized mean framewise displacement, median framewise displacement, maximum framewise displacement, and the percentage of frames exceeding 0.50 mm.
2. Co-registration: Functional images were co-registered to the respective T1 structural image of each subject.
3. Normalization: Spatial normalization to the standard Montreal Neurological Institute (MNI) space.
4. Segmentation: Brain tissue and surgical void segmentation to exclude resected tumor areas from spurious signals.
5. Smoothing: Gaussian kernel (8 mm FWHM) to enhance signal-to-noise ratio.

### 2.6. Activation Score Extraction

At the single-subject level, first-level GLMs were constructed to compare task activation during noun presentation blocks (verb generation) versus rest. The “activation score” for each voxel was defined as the t-value derived from the task-versus-rest contrast.

Feature-Selection Framework. To isolate the ensemble of voxels that captured treatment-related functional change while rigorously preventing information leakage, we implemented a two-stage, nested feature-selection framework executed independently inside each leave-one-subject-out (LOSO) cross-validation fold. All steps were performed with MATLAB R2024a (MathWorks, Natick, MA, USA).

The first stage removes voxels whose direction of change is inconsistent across patients, thus attenuating noise from heterogeneous surgical cavities and variable hemodynamic baselines. The subsequent statistical pruning attenuates redundancy among highly correlated voxels and focuses the classifier on the smallest, most informative multivariate signature of post-radiotherapy activation shift. Together, the stages realize a balance between neurobiological interpretability, dimensionality reduction, and predictive power, conforming to current best practices for machine-learning analyses of pediatric neuro-oncology imaging data.

### 2.7. Feature Selection

#### 2.7.1. Stage 1—Consistency-Aware Searchlight Masking

1. Sign-consistency filter

For every training subject *s* and voxel *v*, we computed the signed activation difference

$$\Delta_{s,v}=T_{s,v}^{TP2}-T_{s,v}^{TP1}$$

where T denotes the first-level task-versus-rest *t*-statistic. All change measures in this study are defined as TP2 minus TP1, so that positive values denote an increase after radiotherapy. A voxel was provisionally retained if at least θ × N_train_ subjects shared the same sign of $\Delta_{s,v}$ (positive *or* negative), where θ is the feature threshold. Six candidate thresholds were specified in advance (θ = 0.55, 0.60, 0.65, 0.70, 0.75, 0.80). Two threshold-selection protocols were applied in parallel to the same pre-specified candidate values, and both results are reported. The nested protocol provides the primary classification estimate. The cohort-wide protocol is reported only as a non-nested threshold-sensitivity analysis and as the basis for the descriptive spatial characterization:

(a) Nested sign-consistency threshold (θ) selection. Rather than selecting a single θ from the complete set of folds, θ was treated as a hyperparameter and re-selected within each fold: within every outer LOSO fold, an inner leave-one-subject-out loop restricted to the 30 training subjects evaluated all six candidates, and the value with the highest inner-loop accuracy was then used to refit the entire pipeline on those 30 subjects before application to the held-out subject. A candidate was eligible only if it produced a non-empty feature set in at least 90% of inner folds; inner folds in which it produced none were scored at chance, and ties were resolved in favor of the more stringent threshold (see Section 2.8).
(b) Cohort-wide sign-consistency threshold (θ) selection. The threshold where maximized mean classification accuracy across folds was selected using all folds (see Section 2.8). This threshold was then applied as a fixed setting to reduce the influence of idiosyncratic voxel-wise changes and to emphasize spatially consistent treatment-associated effects. It should not be interpreted as an externally validated hyperparameter.

2. Searchlight construction

Each sign-consistent voxel served as the center of a spherical searchlight [19] with a kernel of radius 4 voxels. Spheres containing fewer than 25% of the theoretical 257 voxels (i.e., <64 voxels) after the sign filter were discarded to ensure spatial stability. This radius was selected to favor spatially stable, cluster-level multivariate patterns rather than scattered single-voxel features. Because the first-level activation maps were derived from data smoothed with an 8 mm FWHM Gaussian kernel, the searchlight radius was interpreted as operating on spatially smoothed activation fields rather than on independent native-resolution voxels.

3. Sphere ranking

Within each eligible sphere, we computed a robust variant of the voxel-wise Fisher score [20] (signal-to-noise ratio) between TP1 and TP2, in which the squared mean difference and summed variances are replaced by the absolute mean difference and summed standard deviations:

$$F_{v}\;=\frac{\left|\mu_{TP2,v}-\mu_{TP1,v}\right|}{\sigma_{TP2,v}+\sigma_{TP1,v}+\varepsilon },$$

where $\mu_{TPk,v}$ and $\sigma_{TPk,v}$ are the mean and standard deviation of the first-level task-versus-rest t-statistic at voxel v across training samples in timepoint k, and ε is a small positive constant added as a numerical proxy for an infinitesimal offset to prevent division by zero. The Fisher score variant was computed only for voxels within the sphere that also satisfied the sign-consistency mask. The sphere score was defined as the mean of $F_{v}$ across voxels in the sphere. Spheres were ranked by score (descending), and the top 50% were retained.

4. Mask assembly

The union of voxels belonging to the retained spheres constituted the feature mask for that fold. This mask typically encompassed <10% of intracranial voxels, thereby reducing dimensionality by an order of magnitude while preserving spatial contiguity.

All computations in steps 2–4 considered only training data, guaranteeing strict independence from the held-out subject.

#### 2.7.2. Stage 2—Recursive Feature Elimination with Linear Support Vector Machine (SVM)

Within the identified mask described above (see Stage 1), a linear Support Vector Machine (SVM [21,22]; default C = 1) was trained on the concatenated voxel values from the training data (label 0 = pre-irradiation, 1 = post-irradiation). Feature pruning followed a modified recursive feature elimination (RFE) schedule:

1. Weight ranking. Absolute SVM weights were used as a proxy for feature importance.
2. Fractional retention. Predetermined fractions of the current feature set (100%, 75%, 50%, 25%, 10%, 5%) were evaluated non-sequentially. For each fraction, only the highest-ranked voxels were kept.
3. Inner validation. The retained subset was re-evaluated by leave-one-out cross-validation within the training data only, with folds grouped by subject so that the pre- and post-irradiation scans of a training subject were always assigned to the same partition; splitting individual scans rather than subjects would allow a subject’s pre-irradiation scan to inform the classification of that same subject’s post-irradiation scan. The subset achieving the highest inner-loop accuracy was selected (ties resolved by the smallest feature count).
4. Final model. An SVM was retrained on the optimal subset and subsequently applied to the two scans of the held-out subject.

Because both the searchlight mask and the RFE subset were derived exclusively from training data in every LOSO iteration, the procedure avoided circularity (“double dipping”) and minimized information leakage. In the nested-θ analysis, in which the sign-consistency threshold was additionally re-selected inside each outer fold, this yields a fully nested estimate of performance within this single-center cohort. In the cohort-wide fixed-θ analysis, the threshold was selected using all folds, so the resulting accuracy is not a nested estimate and is reported only as a threshold-sensitivity value.

### 2.8. Classification Analysis

We used the selected voxels to define regions of interest (ROIs). We implemented a binary (pre-irradiation versus post-irradiation scans) SVM classification (MATLAB’s fitcsvm, linear kernel). We performed leave-one-patient-out cross-validation [23]:

1. In each fold, the data from one patient (pre- and post-scans) formed the test set, and the remaining 30 patients formed the training set.
2. In each outer leave-one-subject-out fold, the trained linear SVM generated a continuous decision score for the TP2 class for both scans of the held-out participant. The scan with the higher TP2 decision score was assigned to TP2, and the other scan was assigned to TP1. Thus, the two visits of a participant could not receive the same label. Each participant contributed one paired forced-choice binary outcome, which was scored as correct when the true TP2 scan had a higher TP2 decision score than the true TP1 scan. Accordingly, the effective test sample comprised 31 patient-level outcomes rather than 62 independent scan-level outcomes. Sensitivity and specificity were not reported separately because they were identical to paired accuracy by construction.

Statistical significance of the classification accuracy was assessed with a with-in-subject label-swap permutation test. In each of 20,000 permutations, the TP1 and TP2 labels were exchanged independently within each participant, preserving the paired structure and the class balance, and the paired forced-choice accuracy was recomputed.

### 2.9. Cluster-Level PSC Change (ΔPSC) and Statistical Testing

For each cluster with at least 100 voxels (k) derived from the union of voxels selected across leave-one-subject-out folds at a fixed consistency threshold (θ = 0.70, the value most often chosen by the inner loop), we computed mean task-evoked BOLD PSC within the cluster mask for each participant at pre- and post-radiotherapy visits. Because this mask is defined at a single threshold and pooled across folds, the cluster-level analyses that follow are descriptive and are not cross-validated. Within-subject change was defined as $\Delta PSC(k)={PSC(k)}_{\mathrm{TP}2}-{PSC(k)}_{\mathrm{TP}1}$. For each cluster, we tested whether the median ΔPSC(k) differed from zero using a two-sided Wilcoxon signed-rank test. Wilcoxon effect size was quantified as r = |z|/√N, where z is the normal-approximation statistic from the signed-rank test and N is the number of paired observations. *p*-values were adjusted for multiple comparisons within clusters using the Benjamini–Hochberg false discovery rate (FDR) [24], with q < 0.05 considered significant. For all paired comparisons, effects are reported as Hodges–Lehmann median differences with distribution-free 95% confidence intervals derived by inverting the signed-rank test, corroborated by bias-corrected and accelerated bootstrap intervals from 10,000 resamples of participants.

To assess whether clinical and treatment heterogeneity accounted for between-subject differences in ΔPSC, we fit cluster-wise robust linear regression models with ΔPSC as the outcome and fixed effects for posterior fossa syndrome (yes/no), craniospinal irradiation dose (Gy; continuous), tumor location group (midline/intraventricular versus hemispheric/CPA), sex, and age at irradiation (mean-centered). For each covariate, *p*-values were adjusted across clusters using Benjamini–Hochberg FDR (q < 0.05).

### 2.10. Signature Expression and Multiregional Activation-Contrast Score (MACS) Analyses

To bridge multivariate decoding with interpretable effect metrics, we derived two within-subject scalar measures: (i) an out-of-fold classifier signature expression score and (ii) a post hoc descriptive MACS in PSC units.

Signature expression score (SVM decision function). In each LOSO fold, the final linear SVM (trained only on the training subjects, using the searchlight-derived mask and recursive feature elimination subset determined exclusively within the training data) was applied to the held-out subject’s TP1 and TP2 scans to obtain the SVM decision function value, also known as the decision value (DV) [25]:

$$DV=f\left(x\right)=w^{⊤}x+b$$

DVs were oriented so that higher values corresponded to the post-irradiation class (TP2). Within-subject change was defined as $\Delta DV=DV_{\mathrm{TP}2}-DV_{\mathrm{TP}1}$. We tested whether ΔDV differed from zero using a two-sided Wilcoxon signed-rank test and reported effect size $r=\frac{\left|z\right|}{\sqrt{N}}$. For descriptive reporting, we report the percentage of subjects with ΔDV>0. As a nonparametric confirmation, we performed a within-subject label-swap (sign-flip) permutation test on mean(ΔDV) using 20,000 iterations.

Multiregional activation-contrast score (MACS). After identifying the final set of clusters from the multivariate feature-selection results (see above), clusters (k) were partitioned into two sets according to the sign of their cohort median within-subject change ΔPSC: $C_{+}$ (clusters with positive median ΔPSC) and $C_{-}$ (clusters with negative median ΔPSC). We then defined a multiregional activation-contrast score (MACS) for each timepoint (tp) as:

$$MACS\left(tp\right)=\frac{1}{K_{+}}\sum_{k\in C_{+}}PSC_{k}\left(tp\right)-\frac{1}{K_{-}}\sum_{k\in C_{-}}PSC_{k}\left(tp\right)\;,$$

where $PSC_{k}\left(tp\right)$ is the cluster-wise percent signal change at timepoint, while $K_{+}$ and $K_{-}$ are the numbers of clusters in $C_{+}$ and $C_{-}$, respectively. Within-subject change was defined as $\Delta MACS=MACS_{\mathrm{TP}2}-MACS_{TP1}$ and tested against zero using a two-sided Wilcoxon signed-rank test with effect size r and percentage positive ΔMACS. MACS was not designed or tested as an independently validated scoring system. It is a post hoc, cohort-derived descriptive summary in PSC units based on clusters selected from the same dataset and on cluster directionality defined within the same cohort. Therefore, MACS analyses were considered descriptive only and were not used as validation, prediction, or individual-patient risk stratification.

Association between metrics. We assessed concordance between ΔDV and ΔMACS using Spearman’s rank correlation.

Exploratory five-timepoint longitudinal analysis (MACS trajectory). An exploratory analysis evaluated MACS trajectories across TP1–TP5 in the subset of participants with complete five-visit data (n = 8). MACS was computed at each timepoint using the same cluster definition described above. We tested the overall effect of timepoint using a linear mixed-effects model with timepoint as a categorical fixed effect and a random intercept for the subject—a restricted maximum likelihood (REML) approach [26]. Post hoc, we compared each later timepoint (TP2–TP5) against TP1 using paired Wilcoxon signed-rank tests and applied Benjamini–Hochberg FDR correction across these four comparisons. Anatomical labels were assigned using the Automated Anatomical Labeling atlas, and group-mean BOLD percent signal change was summarized using established PSC-based fMRI activation measures [27,28].

Descriptive assessment of treatment heterogeneity. To evaluate whether the patient-level activation-change metric appeared dominated by radiation-dose or radiation-modality subgroups, we generated an exploratory individual patient visualization of ΔMACS by craniospinal irradiation dose and radiation modality. ΔMACS was defined as the TP2 minus TP1 change in MACS for each participant. Radiation modality was categorized as photon, proton, or mixed modality, and CSI dose was treated descriptively as a continuous variable. We assessed the monotonic association between CSI dose and ΔMACS using Spearman’s rank correlation and compared ΔMACS across radiation-modality groups using the Kruskal–Wallis test. Because of the limited sample size and uneven subgroup distributions, these analyses were considered descriptive and were not interpreted as formal subgroup-adjusted inference.

## 3. Results

The median interval between definitive surgery and TP1 fMRI was 28 days (range, 13–44 days). Individual participant intervals are reported in Supplementary Table S1. Head-motion statistics were available for all 31 paired TP2–TP1 scans. Mean framewise displacement was 0.096 ± 0.045 mm at TP1 and 0.153 ± 0.197 mm at TP2; the corresponding medians were 0.082 mm (IQR 0.064) and 0.101 mm (IQR 0.087), respectively. The Hodges–Lehmann median TP2—TP1 difference was 0.023 mm (95% CI 0.002 to 0.052 mm; BCa bootstrap 95% CI 0.003 to 0.052 mm), signed-rank *p* = 0.029, matched-pairs rank-biserial r = 0.45 (95% CI 0.04 to 0.75). Head motion was therefore slightly but reliably higher at TP2, although the upper confidence bound of 0.052 mm is approximately one tenth of the 0.50 mm threshold conventionally used to flag high-motion volumes and an order of magnitude below the voxel dimensions. The TP2 distribution was right-skewed, with a small number of higher-motion scans inflating the parametric mean difference (0.056 mm) relative to the rank-based estimate. Across scans, the average percentage of frames with framewise displacement >0.50 mm was 2.25%.

### 3.1. Primary Analysis: Nested Sign-Consistency Threshold (θ) Selection

Under nested cross-validation the inner loop selected θ = 0.70 in 18 of the 31 outer folds, θ = 0.65 in 9, θ = 0.60 in 3 and θ = 0.80 in 1; the remaining pre-specified candidates were not selected in any fold. The out-of-sample accuracy of the complete pipeline was 71.0% (22 of 31 patients correctly ordered; 95% Clopper–Pearson interval 52.0–85.8%), above the 50% chance level of this paired forced-choice design (one-sided within-subject label-swap permutation test, 20,000 permutations, *p* = 0.015). Additional sensitivity analyses excluding participants with complete mutism or reduced speech, adjusting the first-level models for the six rigid-body motion parameters, using alternative sphere-ranking criteria, and reducing the searchlight radius are summarized in Supplementary Table S2.

### 3.2. Threshold-Sensitivity Analysis: Cohort-Wide Fixed Sign-Consistency Threshold (θ)

**Figure 1:**
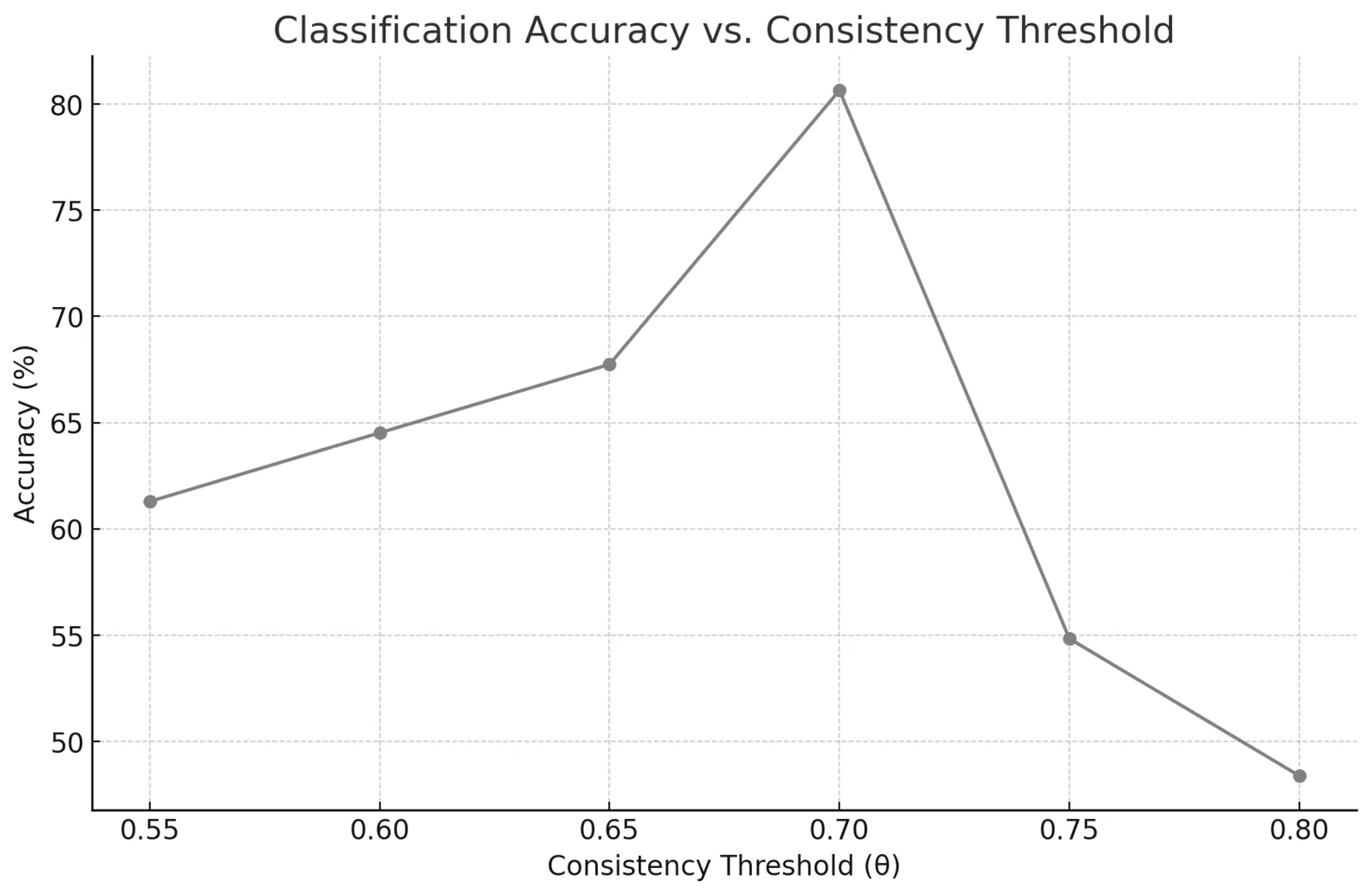
Leave-one-subject-out classification accuracy of a linear SVM separating TP1 (pre-radiotherapy) vs. TP2 (early post-radiotherapy) scans using whole-brain voxel-wise task-versus-rest activation t-maps from silent verb-generation fMRI (n = 31 paired subjects). The consistency threshold (θ) is the minimum fraction of training subjects that must share the same sign of within-subject activation change (Δt = t_TP2 − t_TP1) for a voxel to be retained in the stage-1 sign-consistency filter (followed by searchlight ranking and SVM recursive feature elimination). Accuracy peaked at θ = 0.70 (80.7%).

Applying the same pipeline with fixed θ cohort-wide, LOSO cross-validation revealed a non-monotonic relation between θ and decoding accuracy. Overall cross-validated accuracy increased steadily from θ = 0.55 (61.3%) to θ = 0.70, where it peaked at 80.7% (Figure 1). Beyond this optimum, performance deteriorated sharply: θ = 0.75 yielded accuracy slightly above chance (54.8%), and θ = 0.80 fell to 48.4%.

**Figure 2:**
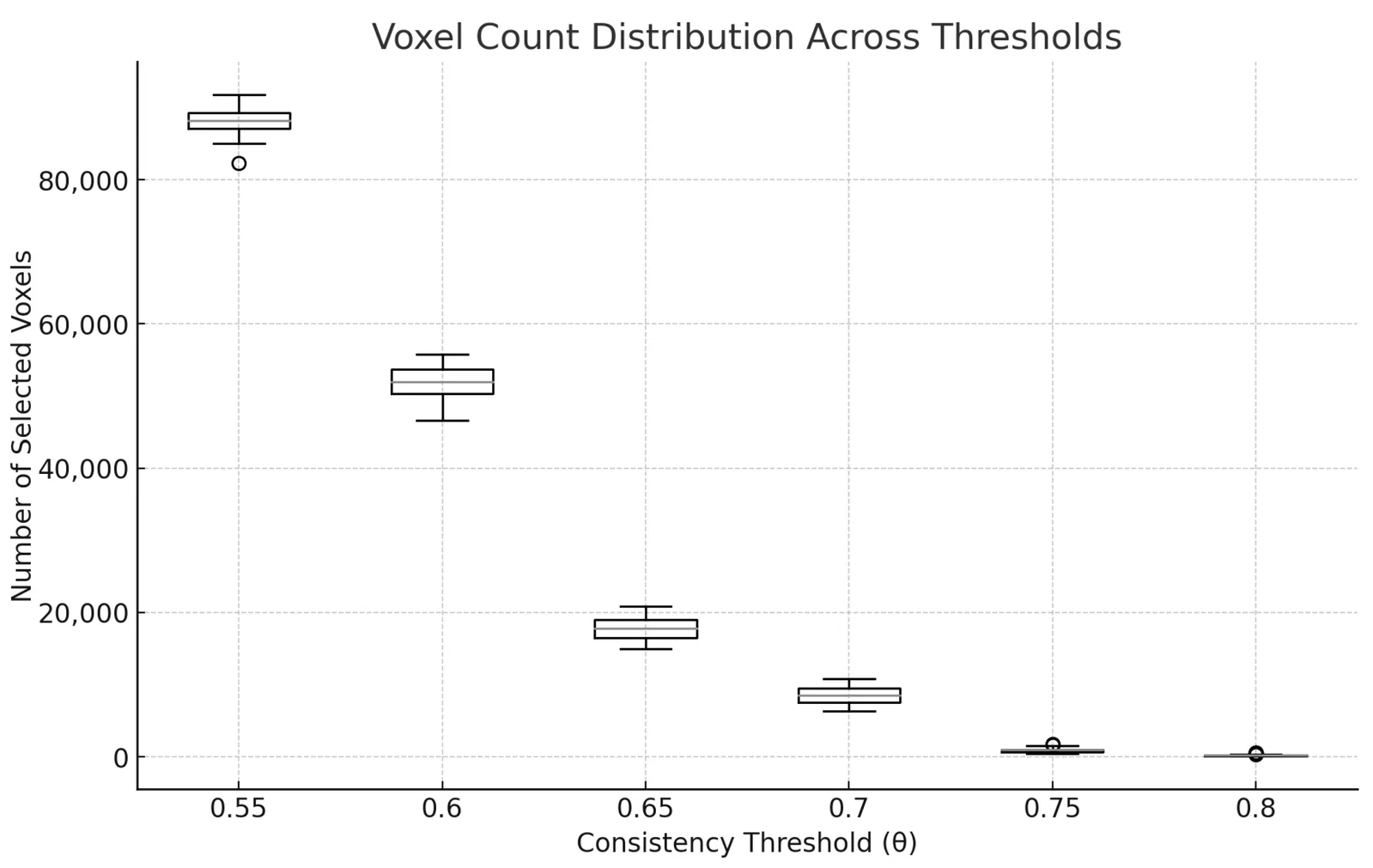
Distribution of the number of voxels entering the classifier at each sign-consistency threshold (θ) across leave-one-subject-out folds. Voxels were retained only if the within-subject activation change (Δt = t_TP2 − t_TP1) had the same sign in at least θ of the training subjects (stage 1), then reduced by searchlight ranking and SVM recursive feature elimination (stage 2). Boxplots summarize per-fold feature counts, illustrating the trade-off between enforcing cross-subject consistency and retaining sufficient features.

Concomitantly, the number of voxels retained after the two-stage feature-selection pipeline collapsed exponentially with more stringent thresholds (Figure 2). Median feature count shrank from ~87 k voxels at θ = 0.55 to ~8 k voxels at θ = 0.70 and to <200 voxels at θ = 0.80. The accuracy peak therefore depended on the balance between group-level consistency (high θ) and information sufficiency (adequate feature dimensionality).

Collectively, these results demonstrate that enforcing a moderate (70%) sign-consistency criterion produced the highest cohort-wide fixed-threshold accuracy in the post hoc threshold-sensitivity analysis, while retaining a neuroanatomically interpretable subset of voxels. At this threshold (θ = 0.70), we further characterized the fixed-threshold model using LOSO and a paired pre-to-post label-swap permutation test (n = 20,000) that preserves the within-subject structure. Observed accuracy under the true labels (80.7%) was compared with the randomized-classification null mean of 50% (95% null confidence interval: 32.26–67.74%). The improvement was significant (one-sided permutation *p* = 0.0004), which indicates that the decision values obtained at this fixed threshold are not attributable to chance labeling. The 80.7% value remains a post hoc, cohort-wide estimate, and the nested estimate reported above (71.0%) is the primary classification result.

### 3.3. Characterization of the Discriminative Activation Signature

**Figure 3:**
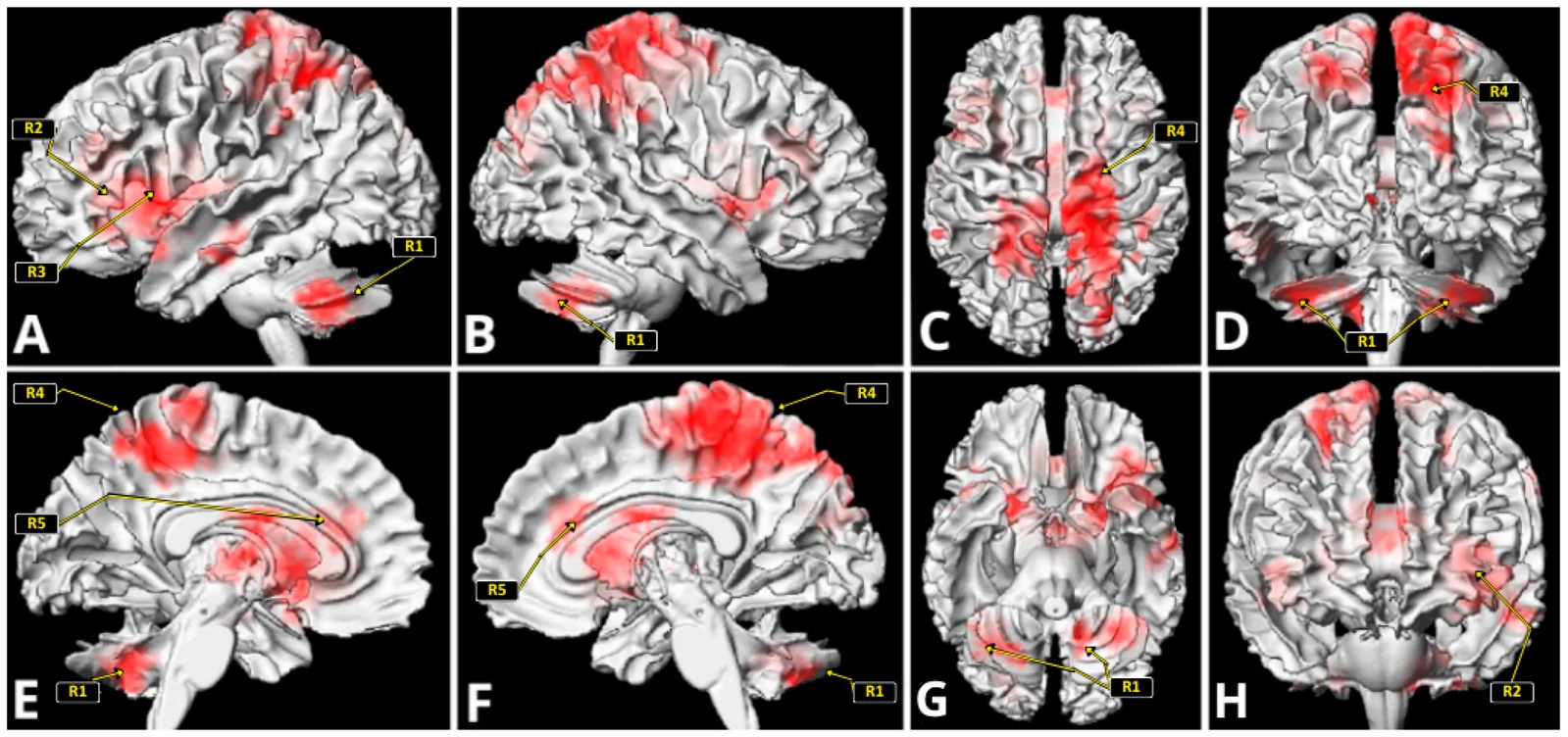
Spatial distribution of informative voxels at the sign-consistency threshold (θ = 0.70), representing both the modal inner-loop threshold and the cohort-wide peak threshold, for the consistency-aware searchlight feature selection. A voxel appears overlaid in red if it was retained as a feature in at least one leave-one-subject-out fold. Panels show 3D brain surface render views ((**A**): left lateral; (**B**): right lateral; (**C**): superior; (**D**): posterior; (**E**): left medial; (**F**): right medial; (**G**): inferior; (**H**): anterior). Posterior (**D**), inferior (**G**), lateral (**A**,**B**), and medial (**E**,**F**) views emphasize the bilateral cerebellum [R1], highly exposed during craniospinal and posterior-fossa irradiation. Left lateral (**A**) and anterior (**H**) view show left inferior frontal gyrus [R2] (pars opercularis, triangularis; Broca’s region), key for lexical-semantic retrieval [29]. Left lateral (**A**) view also shows anterior insula [R3] key for articulatory planning [30]. Superior (**C**), posterior (**D**), and medial (**E**,**F**) views highlight the superior parietal lobule, precuneus, and postcentral regions [R4], which are phonological and attentional components of verb generation [12,31,33]. Medial views (**E**,**F**) also demonstrate the anterior cingulate/medial frontal cortex [R5] region implicated in speech initiation, response selection, attentional control, and performance monitoring during word production [32].

Since the sign-consistency threshold θ = 0.70 was the value most often selected by the inner loop (18 of 31 folds) of the nested model as the modal threshold, and also coincided with the peak of the cohort-wide fixed-threshold model as the performance optimal, we descriptively characterized the features selected at θ = 0.70 as a neuroanatomically interpretable pattern (Table 2) encompassing canonical language and control regions [12,29,30,31,32,33] along with cerebellar territories [13] commonly implicated in language–motor integration (Figure 3).

Within-subject ΔPSC analyses revealed statistically robust parietal changes. The “right precuneus/superior parietal/paracentral/postcentral” cluster showed attenuation of task-negative response (mean ΔPSC = +0.323; Hodges–Lehmann median difference +0.276, 95% CI [0.105, 0.471], BCa bootstrap 95% CI [0.116, 0.463]; Wilcoxon signed-rank *p* = 0.0017, q = 0.006, r = 0.55). The “left supramarginal/inferior parietal” cluster showed increased activation (mean ΔPSC = +0.338; Hodges–Lehmann median difference +0.278, 95% CI [0.130, 0.511], BCa bootstrap 95% CI [0.140, 0.511]; Wilcoxon signed-rank *p* = 0.00034, q = 0.002, r = 0.61). The “left cerebellar” cluster showed reduced activation (mean ΔPSC = −0.179; Hodges–Lehmann median difference −0.185, 95% CI [−0.331, −0.030], BCa bootstrap 95% CI [−0.318, −0.039]; Wilcoxon signed-rank *p* = 0.021, q = 0.049, r = 0.42). Other clusters (including right cerebellum; left inferior frontal gyrus, insula, and basal ganglia; anterior cingulate; and left middle temporal gyrus) showed negative mean ΔPSC but did not survive FDR correction (lowest q = 0.145). In sensitivity analyses adjusting for covariates, ΔPSC was not significantly associated with posterior fossa syndrome history, craniospinal irradiation dose, tumor location group, sex, or age at irradiation, either in uncorrected tests (all *p*-values > 0.05) or after false discovery rate (FDR) correction (all q-values > 0.05).

In the cerebral cortex, decreased activation was observed in the left inferior frontal gyrus (pars opercularis/triangularis) and the anterior insula, regions implicated in lexical retrieval and articulatory planning. Medial frontal contributions included the anterior cingulate cortex; parietal contributions included the superior parietal lobule, consistent with attentional control, phonological working-memory support, response selection, and performance monitoring. Parietal engagement comprised superior parietal lobule and precuneus, with extension into the paracentral/postcentral cortex, aligning with phonological working memory and attentional components of verb generation.

Subcortically, there was robust bilateral involvement of cerebellar Crus I/II and lobules VI–VIII, which are territories of the cerebellar–cerebral language loop that are highly exposed during craniospinal and posterior-fossa irradiation [13].

Taken together, these spatial findings support the interpretation that post-irradiation reductions in task-related activation occur within a cerebello-frontal–temporal network subserving language production and cognitive control, and that a moderate sign-consistency criterion preferentially captures these treatment-sensitive regions.

Signature expression score (SVM decision function). Out-of-fold SVM DV, oriented so that higher values corresponded to the TP2 (post-irradiation) class, showed a consistent within-subject shift from TP1 to TP2. Across 31 subjects, $\Delta DV=DV_{\mathrm{TP}2}-DV_{TP1}$ was positive in 25 of 31 (80.6%). Median ΔDV = 0.846 (IQR 1.598) and mean ΔDV = 0.953. The paired shift was significant by Wilcoxon signed-rank (*p* = 0.00065; r = 0.612) and remained significant in a within-subject label-swap (sign-flip) permutation test on mean(ΔDV) (two-sided *p* = 0.0003; 20,000 iterations).

Multiregional activation-contrast score (MACS). The PSC-unit MACS, defined as the mean PSC across clusters with positive cohort median ΔPSC($C_{+}$) minus the mean PSC across clusters with negative cohort median ΔPSC($C_{-}$), increased from TP1 to TP2 in 30/31 (96.8%) subjects. In this cohort, $C_{+}$ comprised clusters on parietal regions, while $C_{-}$ comprised clusters on cerebellar, frontal, insula, and cingulate regions (see Table 2 for reference values). Within-subject change $\Delta MACS=MACS_{TP2}-MACS_{\mathrm{TP}1}$ had median 0.364 (IQR 0.358) and mean 0.453, with a Hodges–Lehmann median difference of +0.377 (95% CI [0.254, 0.540]; BCa bootstrap 95% CI [0.259, 0.544]). Because clusters were assigned by the sign of their own cohort, median ΔPSC and MACS were then evaluated on the same participants. This interval describes the precision of a post hoc descriptive summary rather than independent evidence of change. Association between metrics. ΔMACS correlated with ΔDV(Spearman ρ = 0.514, *p* = 0.0031), indicating that the clinically interpretable activation contrast captured a substantial portion of the multivariate signature expression.

**Figure 4:**
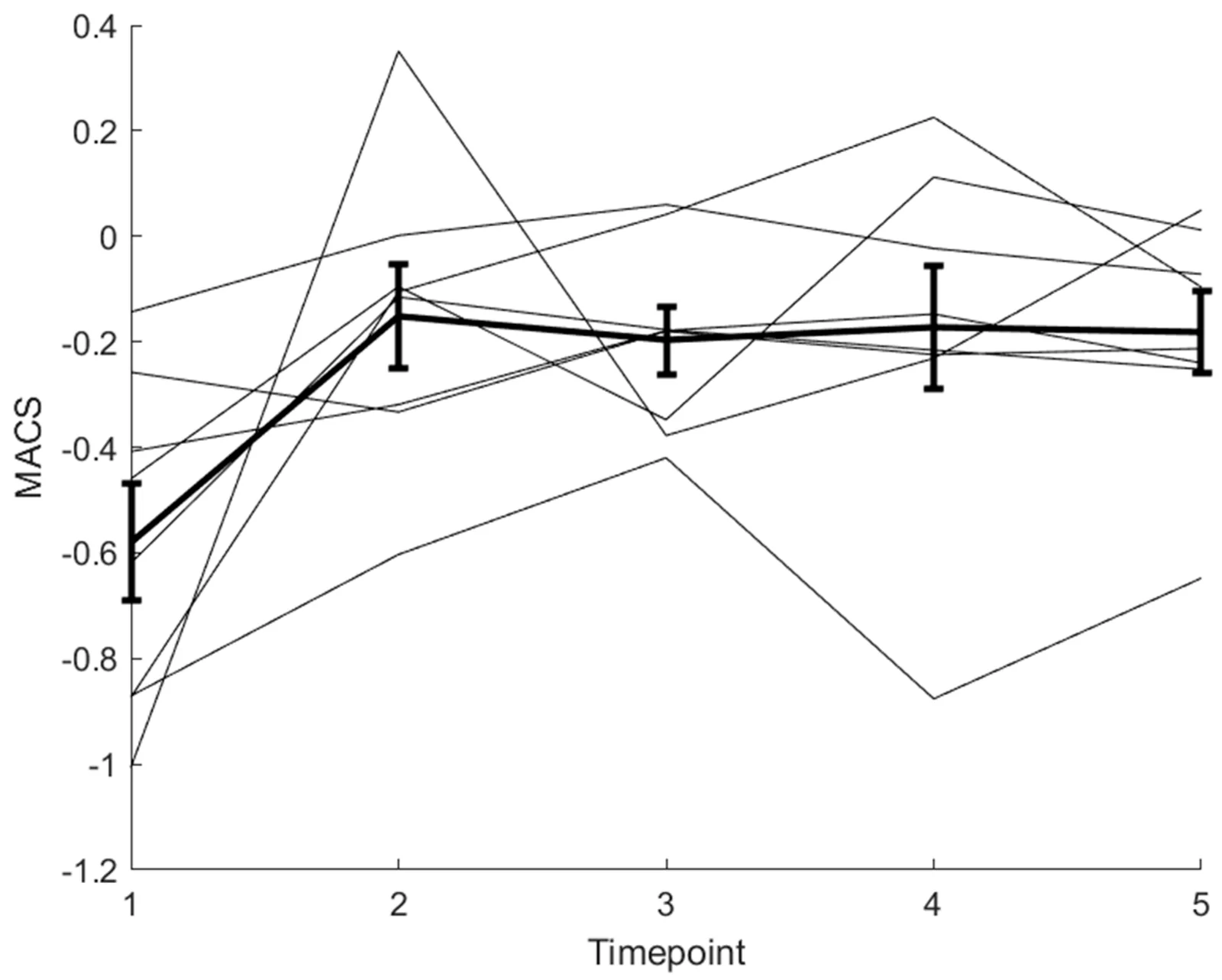
Descriptive exploratory multiregional activation-contrast score (MACS) trajectory across TP1–TP5 (n = 8). Thin lines show individual participants; the thick line shows mean ± SE. Timepoints are protocol-defined: TP1—pre-irradiation; TP2—post-irradiation/pre-chemotherapy; TP3—post-chemotherapy; TP4—3-month follow-up after TP3; TP5—6-month follow-up after TP4. MACS (in percentage signal change units) increased from TP1 to TP2 and remained elevated through TP5, consistent with a durable multiregional activation shift beyond the immediate post-irradiation window.

Exploratory five-timepoint MACS trajectory. In the subset of participants with complete five-visit data (n = 8), MACS trajectories are shown in Figure 4 as a descriptive visualization only. MACS showed a significant overall timepoint effect (linear mixed-effects model with timepoint as categorical fixed effect and subject random intercept: *p* = 0.0014). Relative to TP1 (pre-irradiation), MACS was higher at TP2 (post-irradiation/pre-chemotherapy; median ΔMACS = 0.316; *p* = 0.016; 7/8 positive), TP3 (post-chemotherapy; median ΔMACS = 0.336; *p* = 0.008; 8/8 positive), TP4 (3-month follow-up after TP3; median ΔMACS = 0.332; *p* = 0.016; 7/8 positive), and TP5 (6-month follow-up after TP4; median ΔMACS = 0.295; *p* = 0.008; 8/8 positive). All four comparisons remained significant after FDR correction (q = 0.016). The trajectory showed an early upward shift from TP1 to TP2 that persisted through TP5 (Figure 4).

**Figure 5:**
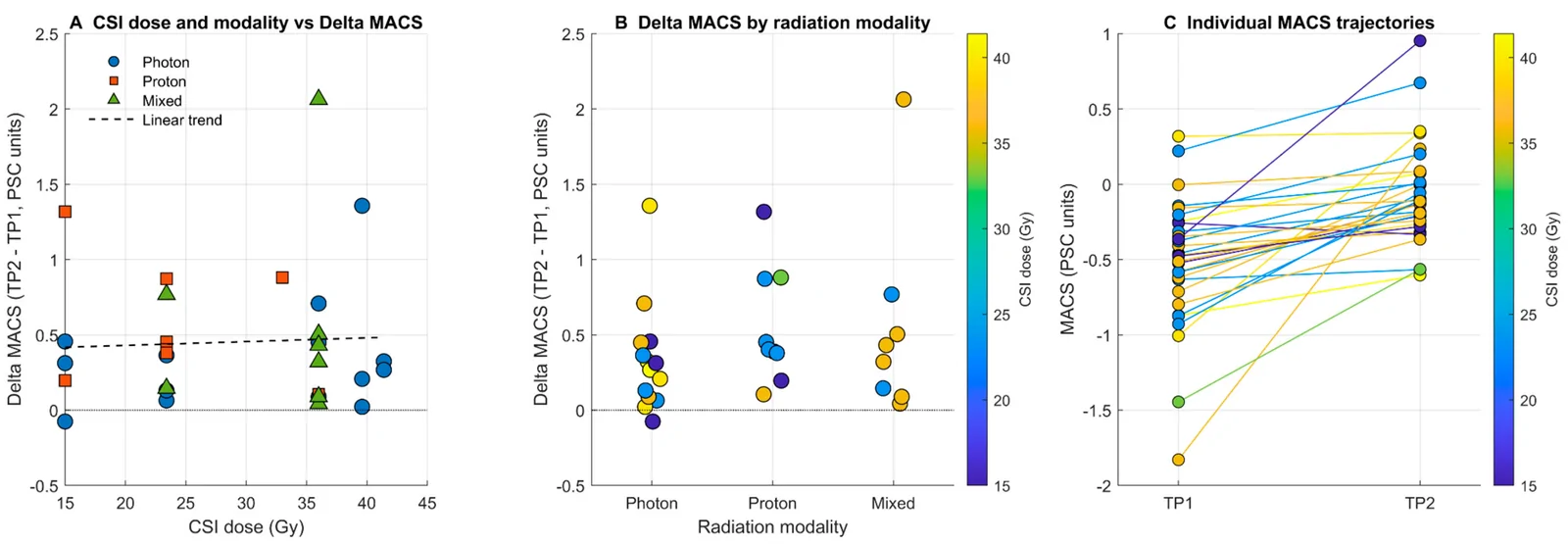
Descriptive assessment of individual TP1–TP2 multiregional activation-contrast score (MACS) change by craniospinal irradiation dose and radiation modality. (**A**) ΔMACS, defined as a TP2-minus-TP1 multiregional activation-contrast score, plotted against craniospinal irradiation dose. Points represent individual patients and are coded by radiation modality; the dashed line shows the descriptive linear trend. (**B**) ΔMACS displayed by radiation modality, with individual points color-coded by craniospinal irradiation dose. (**C**) Individual paired TP1–TP2 MACS changes, color-coded by craniospinal irradiation dose.

Descriptive visualization of treatment heterogeneity. To assess whether the patient-level activation-change metric appeared dominated by radiation dose or modality, we added a descriptive individual-patient visualization of ΔMACS by craniospinal irradiation dose and radiation modality (Figure 5). Across the full cohort, ΔMACS had a mean ± SD of 0.453 ± 0.458 PSC units and a median [IQR] of 0.364 [0.358]. There was no apparent monotonic association between CSI dose and ΔMACS (Spearman ρ = −0.057, *p* = 0.7619), and ΔMACS did not differ significantly across radiation modalities in this descriptive analysis (Kruskal–Wallis *p* = 0.2529). Median ΔMACS values were 0.290 for photon-treated patients, 0.403 for proton-treated patients, and 0.377 for patients receiving mixed-modality treatment. These exploratory visualizations did not suggest that the MACS shift was driven exclusively by a single CSI-dose level or radiation-modality subgroup.

## 4. Discussion

Our findings corroborate the established concern that pediatric radiotherapy can lead to alterations in functional brain activation, potentially leading to cognitive deficits [34].

The machine-learning pipeline identified a distributed signature spanning cerebellar, frontal, medial, and parietal regions. When within-subject change in these clusters was quantified using ΔPSC (TP2 − TP1), the most statistically robust effects were parietal: attenuation of pre-treatment task-negative response in the right precuneus and superior parietal cluster and increased activation in the left supramarginal and inferior parietal cluster (Wilcoxon signed-rank q < 0.01; 71–81% of participants showing increases). In contrast, mean decreases were observed in cerebellar and left fronto-temporal, insular, cingulate clusters. Of these, only the left cerebellar cluster survived FDR correction (q = 0.049); the remaining clusters showed substantial individual variability and did not survive correction. Importantly, lack of FDR significance for individual clusters does not imply irrelevance for decoding: multivariate classifiers can leverage weak and/or heterogeneous regional effects when they combine with other features and covariance structure. Accordingly, we interpret these cerebello–frontoinsular and cingulate regions as components of a distributed multivariate signature rather than as stand-alone univariate effects.

Reductions in task-evoked BOLD response in classical cortical language regions, particularly the inferior frontal gyrus and the left middle temporal gyrus, may reflect less efficient recruitment of controlled lexical-semantic retrieval and selection processes during covert verb generation [29,30]. Given potential post-radiotherapy alterations in neurovascular coupling, these changes may also partly reflect hemodynamic responsivity rather than purely neuronal effects. Moreover, these findings indicate that treatment-related changes in task-evoked activation during silent verb generation are not confined to a single perisylvian “language center,” but instead involve a distributed network spanning frontal, temporal, parietal, and cerebellar territories commonly implicated in lexical retrieval, articulatory planning, phonological buffering, and attentional control [30]. Because these regional decreases showed substantial inter-individual variability and, apart from the left cerebellar effect, did not survive FDR correction, they are best interpreted as components of the multivariate signature rather than as stand-alone univariate effects.

At the modal and performance-optimal sign-consistency threshold (θ = 0.70), features with the greatest discriminative value localized to the left inferior frontal gyrus (pars opercularis and triangularis) and anterior insula, medial-frontal control regions including anterior cingulate, dorsal parietal and precuneus regions with extension into paracentral/postcentral cortex, and bilateral cerebellar Crus I/II and lobules VI–VIII.

This anatomy is compatible with the cerebellar–cerebral language loop and suggests that radiation exposure, particularly to posterior fossa structures and their thalamo-cortical projections, may perturb an extended production and control circuit rather than a narrow cortical module. Notably, the patterned (but individually variable) decrease in the cerebellar response during the silent verb-generation task for patients after radiotherapy aligns with growing evidence that cerebellar Crus I/II and lobules VI–VIII contribute to higher cognitive operations, including language production, articulation coordination, and executive sequencing [35,36,37].

The increased engagement in the left supramarginal gyrus and inferior parietal lobule likely reflects a greater reliance on dorsal-stream phonological mechanisms to support covert production demands when core production and control regions (left inferior frontal gyrus, insula, anterior cingulate cortex, cerebellum) show reduced task-evoked responses. The left supramarginal gyrus and inferior parietal lobule are classically implicated in phonological storage [38] and sublexical grapheme–phoneme coding [39], with projections via superior longitudinal fasciculus [40] and arcuate fasciculus [41] to the premotor cortex and inferior frontal gyrus supporting articulatory rehearsal during covert speech. Because this effect persists when pooling auditory and visual cueing and primary sensory cortices are not consistent contributors, it is less likely to be sensory-driven; instead, it appears modality-independent, indexing increased phonological working-memory load. Although overt task performance was not directly measured during scanning, this pattern is compatible with possible compensatory recruitment of parietal phonological buffering systems. Clinically, this motivates targeting phonological rehearsal strategies and working-memory scaffolds to harness supramarginal gyrus and inferior parietal lobule recruitment, consistent with prior trials of cognitive working-memory training in pediatric brain tumor survivors [42].

The shift from task-negative at pre-irradiation toward a near-zero response post-irradiation in the right hemisphere indicates attenuation of task-negative activity in a large cluster overlapping medial parietal (precuneus) and dorsal parietal and somatomotor territories. This pattern is consistent with weaker suppression of default-mode processes at medial parietal regions (precuneus) during task engagement, which has been linked to reduced processing efficiency and greater intrusion of internally directed cognition in demanding tasks [43]. In addition, reduced precuneus deactivation under demand has been interpreted as reflecting increased monitoring and control demands in some contexts [44]. However, in clinical cohorts, it is frequently discussed in terms of inefficient task engagement or reduced default-mode suppression. At the same time, because the cluster extends into superior parietal, paracentral, and postcentral cortices, the net ΔPSC may also reflect greater reliance on attention and sensorimotor/phonological rehearsal mechanisms (e.g., covert articulatory or somatosensory imagery) to stabilize retrieval when frontal–cerebellar components are weakened [45]. Notably, these ΔPSC shifts were not significantly explained by posterior fossa syndrome history, craniospinal irradiation dose, tumor location group, sex, or age at irradiation in covariate sensitivity analyses, although larger cohorts will be required to test moderation effects with adequate power.

Beyond classification accuracy, the out-of-fold signature expression decision values provide a patient-level measure of the distributed multivariate effect, showing a consistent TP1-to-TP2 shift in most participants. Complementarily, the PSC-unit MACS (mean of the parietal clusters minus the mean of the remaining clusters) showed a highly consistent increase, supporting a robust rebalancing toward parietal recruitment during silent verb generation after radiotherapy. The correlation between these metrics suggests that a simple contrast in PSC units captures meaningful variance in the multivariate signature while remaining clinically interpretable.

TP2 scans showed reduced task-evoked activation in the supragenual anterior cingulate cortex, a medial frontal region implicated in performance monitoring, attentional control, and selection during speech production [32,46,47]. However, task fMRI alone cannot determine whether this observed change reflects direct tissue injury, vascular effects, or compensatory reorganization. A potential concern in early post-RT fMRI is that observed differences could reflect transient physiological or behavioral effects rather than durable reorganization. In the complete five-visit subset (n = 8), MACS increased at TP2 (the only post-irradiation/pre-chemotherapy follow-up) and remained elevated at later protocol-defined visits through TP5, including post-chemotherapy and 6-month follow-up (omnibus LME *p* = 0.0014; all TP2–TP5 vs TP1 q = 0.0156). Although later phases include additional exposure (chemotherapy), the persistence of the activation-contrast supports the interpretation that the early post-irradiation shift is not purely transient and may reflect a stable change in the balance between parietal engagement and cerebello–frontoinsular and cingulate components of the signature. The exploratory TP1–TP5 visualization should be interpreted cautiously because it included only eight patients. They are retained only as descriptive, hypothesis-generating observations to motivate larger longitudinal studies with complete follow-up and neurocognitive outcomes.

Regarding practical implications, the out-of-fold SVM DV (signature expression) and the PSC-unit MACS provide complementary summaries of the multivariate activation pattern and may be useful as candidate longitudinal research measures. In this cohort, both measures showed a consistent within-subject post-irradiation shift (ΔDV > 0 in 25 of 31 participants; ΔMACS > 0 in 30 of 31, Hodges–Lehmann median difference +0.377, 95% CI [0.254, 0.540]). Both metrics derive from the cohort-wide fixed-θ model and from clusters defined in the same participants, so they summarize the selected pattern descriptively rather than providing independent confirmation of the nested classification result. Future prospective studies should test whether longitudinal changes in ΔDV, MACS, or regional ΔPSC predict subsequent language, working-memory, or executive-function outcomes and whether they add value beyond standard clinical and structural imaging measures.

However, clinical scalability will depend on streamlining acquisition (brief, motion-robust paradigms feasible across ages), automating preprocessing, and demonstrating cross-scanner reproducibility and incremental value beyond standard surveillance MRI; therefore, near-term use is most realistic within prospective trials or targeted high-risk subgroups. Finally, the anatomical pattern points to plausible targets for early intervention (e.g., language therapy emphasizing lexical retrieval and phonological rehearsal; supports for verbal attention and working memory; school accommodations that offload working-memory demands) and, cautiously, motivates future investigation of neuromodulatory strategies [48] aimed at frontal–cerebellar circuitry.

Methodologically, the non-monotonic accuracy profile across thresholds clarifies an important trade-off in feature selection for pediatric longitudinal fMRI: as θ increases, voxels with inconsistent group-level signs are excluded, improving noise robustness; yet overly stringent thresholds collapse feature dimensionality and remove informative but moderately variable regions. Both the peak and the mode at θ = 0.70 likely reflect a sweet spot balancing group-level reliability with sufficient coverage to capture treatment-sensitive signal across cerebello-frontal pathways. LOSO cross-validation provides evidence of above-chance discrimination within this internally cross-validated single-center cohort. Under nested cross-validation the inner loop selected θ = 0.70 in 18 of the 31 outer folds, and the accuracy of the complete pipeline was 71.0% (95% Clopper–Pearson interval 52.0–85.8%), above the 50% chance level of this paired forced-choice design (one-sided within-subject label-swap permutation test, 20,000 permutations, *p* = 0.015). In the non-nested fixed θ = 0.70 analysis, we also compared the LOSO performance to a randomized baseline using a paired TP1-to-TP2 label-swap permutation test (n = 20,000) that preserves within-subject structure, confirming that the observed 80.7% accuracy was significantly above chance (null mean 50%, 95% CI [32.26%, 67.74%], one-sided *p* = 0.0004). Sensitivity analyses showed that exclusion of participants with post-operative speech impairment and first-level adjustment for motion produced accuracy close to the primary estimate, whereas alternative ranking criteria and the smaller three-voxel searchlight yielded lower accuracy (Supplementary Table S2). Accordingly, the direction of the classification effect was broadly preserved, but its magnitude should be interpreted as somewhat dependent on analytic specification.

The silent verb-generation task may provide a research-sensitive probe of post-treatment changes in language-related activation that are not readily captured by structural imaging alone, particularly given prior evidence that craniospinal irradiation is associated with atypical white-matter development in pediatric brain tumor survivors [49]. However, because the present study did not link fMRI metrics to concurrent or subsequent neurocognitive outcomes, these activation changes should not be interpreted as clinical indicators of later impairment. Future interventional studies could test whether language therapy, lexical-retrieval support, phonological working-memory strategies, structured working-memory training, and school accommodations that reduce verbal working-memory load are associated with improved cognitive outcomes or normalization of task-evoked activation patterns [43].

Moreover, contemporary radiotherapy approaches, particularly proton radiotherapy, can reduce normal-tissue exposure in pediatric medulloblastoma while maintaining survival outcomes comparable to photon-based treatment, supporting efforts to limit dose to vulnerable neural structures and reduce late treatment-related sequelae [50]. Substructure-informed planning may be especially important because brain substructure dose has been associated with cognitive outcomes in children treated for medulloblastoma [4]. This rationale underscores an urgent need for integrative protocols combining imaging-based risk stratification with biologically informed therapeutic strategies.

Regarding limitations that warrant consideration, the typical sample sizes in pediatric oncology fMRI studies reduce power for detailed region-by-region analysis. Additionally, treatment heterogeneity remains an important limitation. Participants received different craniospinal irradiation doses and radiation modalities, which may differ in normal-tissue dose distributions and biological effects. Although the descriptive patient-level visualization in Figure 5 did not suggest that ΔMACS was driven exclusively by a single CSI-dose level or radiation-modality subgroup, the cohort was not powered for dose- or modality-stratified inference. Therefore, residual confounding by treatment characteristics cannot be excluded.

Motion and arousal remain potential confounds in child cohorts. Although median framewise displacement was low at both timepoints, TP2 scans showed somewhat higher and more variable motion than TP1 scans. Therefore, residual motion-related effects cannot be fully excluded and should be considered when interpreting longitudinal BOLD differences.

Because responses were generated silently, trial-level behavioral accuracy could not be measured during scanning. This limits certainty about trial-by-trial task engagement and performance, particularly in children and clinically vulnerable patients, and therefore constrains interpretation of whether observed BOLD differences reflect treatment-associated activation changes, differences in compliance, or variation in cognitive effort. Due to the absence of neurocognitive outcome analyses, the observed TP2–TP1 fMRI changes cannot be interpreted as evidence of cognitive decline, compensation, or clinical risk in individual patients.

A key limitation is that TP2 was the second exposure to the silent verb-generation paradigm. Without a healthy or non-irradiated patient control group scanned over the same interval, we cannot fully distinguish radiotherapy-associated BOLD changes from practice effects, habituation, reduced novelty, altered arousal, postsurgical recovery, or developmental change. A test–retest study of language fMRI supports broad reproducibility of language lateralization, particularly for verb-generation paradigms, but also indicates that activation volume and spatial extent are less reproducible than laterality measures and should be interpreted cautiously in longitudinal studies [51]. Therefore, the TP1–TP2 changes observed here should be interpreted as early post-treatment-associated activation shifts rather than definitive evidence of radiation injury alone. Future studies should include non-irradiated test–retest controls or repeated pre-radiotherapy scans to quantify task-familiarity effects directly.

Although the classifier was tuned and evaluated with LOSO cross-validation, an external test set and prospective acquisition would strengthen claims of generalizability. LOSO cross-validation provides an internal estimate of model performance within this single-center cohort, but it is not equivalent to an external validation set. The paired label-swap permutation test evaluates whether the observed TP2–TP1 label structure exceeds chance within this dataset; it does not establish cross-site, cross-scanner, or independent-cohort generalizability. Consequently, the classifier and PSC-unit summary measures should be considered discovery-stage tools pending prospective validation in independent cohorts with harmonized acquisition and neurocognitive outcomes.

The searchlight radius also limits fine-grained anatomical specificity. Although the 4-voxel radius was used to improve spatial stability in smoothed pediatric fMRI data and to capture coherent multivariate patterns rather than isolated voxels, it may allow neighboring anatomical territories to contribute to the same searchlight, particularly in compact structures such as the cerebellum. Therefore, cerebellar findings should be interpreted as reflecting broader cerebellar–cerebral language network involvement rather than precise lobule-specific localization. The consistency threshold θ = 0.70 should also be interpreted cautiously. Because this threshold was not validated in an independent cohort, the resulting classification accuracy may be optimistic. Therefore, θ = 0.70 should be viewed as an exploratory analysis setting for the present cohort rather than as an established optimal parameter for future application. The Fisher-score sphere-ranking step may be sensitive to outlying activation values, particularly because each outer training fold contained 30 participants.

While task fMRI identifies treatment-sensitive regions, it does not by itself specify whether reduced activation reflects neuronal inefficiency, compensatory reorganization, or vascular changes. In addition, although signature expression scores were computed out of fold, the spatial clusters used to construct MACS and their positive/negative grouping were derived from the same cohort. MACS should therefore be considered a descriptive cohort-derived summary rather than an externally validated biomarker. Replication in independent samples and association with prospective neurocognitive outcomes are needed before MACS can be interpreted as a generalizable or clinically useful imaging measure.

This approach could be refined by integrating additional features, such as diffusion tensor imaging (DTI) metrics, resting-state connectivity, or volumetric measures, to create a more comprehensive biomarker panel [23,52]. Such biomarkers, once validated, could inform early interventions (e.g., cognitive rehabilitation, pharmacological approaches) to ameliorate or prevent significant functional changes [42,53]. The same multimodal biomarker framework could also provide mechanistic readouts and sensitive endpoints for evaluating neuroprotective agents, such as memantine, by quantifying whether treatment preserves task-evoked network engagement and white-matter integrity over time.

Future work should integrate diffusion metrics, resting-state connectivity, and volumetry with task activation to sharpen mechanistic interpretation and improve predictive accuracy. Dose-mapping against discriminative features could clarify dose–response relationships along cerebello–thalamo–cortical tracts. Longitudinal trajectories spanning acute, subacute, and late effects, paired with language and cognitive assessments, will be essential for linking imaging biomarkers to functional outcomes and for personalizing supportive care. Importantly, this framework could also serve as an objective endpoint for intervention trials by testing whether cognitive training and rehabilitation are accompanied by pre- to post-intervention changes (normalization or strengthening) in functional connectivity and task-evoked engagement within the identified circuit, and whether those neural shifts track behavioral gains.

## 5. Conclusions

In this single-center pilot cohort, a fully nested paired leave-one-subject-out analysis identified an above-chance TP1-to-TP2 ordering signal in silent verb-generation fMRI, correctly ordering 22 of 31 participant pairs (71.0%). Patient-level summary metrics derived from the cohort-wide fixed-θ model described the same effect descriptively: decision values exhibited within-subject shift in 25 of 31 participants (median ΔDV = 0.846).

The PSC-unit multiregional activation-contrast score increased in 30/31 subjects (median ΔMACS = 0.364; Hodges–Lehmann median difference +0.377, 95% CI [0.254, 0.540]). In an exploratory TP1-to-TP5 subset (n = 8), MACS increased after irradiation (TP2, prior to chemotherapy) and remained elevated post-chemotherapy and through 6-month follow-up (LME *p* = 0.0014; TP2–TP5 vs. TP1 q = 0.0156), supporting durability beyond the immediate post-irradiation window. The cohort-derived MACS analysis was retained only as a descriptive PSC-unit summary of this internally selected pattern and should not be interpreted as a validated scoring system, biomarker, or clinical predictor. These descriptive cohort-derived maps implicated distributed cerebellar, frontal, temporal, cingulate, and parietal regions.

Together, these findings provide quantitative imaging observations that warrant prospective validation in relation to longitudinal language and cognitive outcomes. Because the study lacked an external validation cohort, a non-irradiated test–retest control group, concurrent task-performance measures, and longitudinal neurocognitive correlations, the findings do not establish radiotherapy-specific causation, clinical prediction, compensation, or durability. Prospective multi-site studies with harmonized acquisition, motion-robust analysis, appropriate test–retest controls, radiation dose mapping, and longitudinal neurocognitive outcomes are required.

## Figures and Tables

**Table 1:** Demographic and clinical characteristics of the medulloblastoma cohort (n = 31).

Participant Demographic and Clinical Characteristics	Overall (n = 31)
Age at irradiation, years	14.1 ± 4.7
Sex, n (%)	
Male	18 (58.1)
Female	13 (41.9)
Posterior fossa syndrome (PFS) history, n (%)	
Yes	8 (25.8)
No	23 (74.2)
Immediate post-operative neurologic categories, n (%)	
Minimal or no neurological symptoms	22 (71.0)
Complete mutism—PFS	3 (9.7)
Reduced speech—PFS	5 (16.1)
Severe ataxia	1 (3.2)
Tumor location, n (%)	
Midline/intraventricular	21 (67.7)
Hemispheric/Cerebellopontine angle	10 (32.3)
Craniospinal irradiation dose, n (%)	
15 Gy	5 (16.1)
23.4 Gy	10 (32.3)
33 Gy	1 (3.2)
≥36 Gy	15 (48.4)
Primary tumor bed boost dose, n (%)	
51.0 Gy	6 (19.4)
54.0 Gy	24 (77.4)
59.4 Gy	1 (3.2)
Radiation modality, n (%)	
Photon	14 (45.2)
Proton	9 (29.0)
Mixed	8 (25.8)

**Table 2:** Cluster-wise changes in the silent verb generation task-evoked blood oxygen level-dependent (BOLD) signal. Clusters were defined as the union of voxels selected by a sign consistency-based machine-learning model across leave-one-subject-out (LOSO) folds at a fixed threshold of θ = 0.70; only components with ≥100 voxels are reported. For each cluster, the center of gravity is given in MNI coordinates, and anatomical descriptors are provided using the full region names from the Automated Anatomical Labeling [27] atlas (AAL). Group-mean BOLD percent signal change [28] (PSC) is reported for TP1 (pre-radiotherapy) and TP2 (post-radiotherapy) visits. ΔPSC denotes ‘TP2–TP1’; arrows indicate the direction of change (↑ increase, ↓ decrease). Cluster size is the number of voxels in the connected component.

Cluster Labels	Hemisphere	Gravity Center	Cluster Size	Mean PSC		ΔPSC	Direction
(AAL)		(MNI)	(Voxels)	(TP1)	(TP2)	(TP2 − TP1)	
Cerebellum (Crus I, Lobule VIII, Lobule VI, Crus II)	R	30, −63, −39	542	0.371	0.293	−0.078	↓
Cerebellum (Crus I, Lobule VIII, Lobule VI, Crus II)	L	−26, −58, −39	940	0.294	0.115	−0.179	↓
Middle Temporal Gyrus	L	−55, −15, −14	280	0.179	0.133	−0.046	↓
Insular Cortex, Putamen, Inferior Frontal Gyrus (triangular part)	L	−12, 8, 5	5134	0.515	0.381	−0.134	↓
Anterior Cingulate Cortex (Supragenual)	Midline	1, 32, 22	1076	0.345	0.205	−0.140	↓
Precuneus, Superior Parietal Lobule, Paracentral Lobule, Postcentral Gyrus	R	9, −44, 56	7633	−0.353	−0.030	0.323	↑
Supramarginal Gyrus, Inferior Parietal Lobule	L	−62, −43, 37	181	−0.001	0.337	0.338	↑

## Data Availability

The data presented in this study is available on request from the corresponding author due to patient privacy, institutional review board restrictions, and data-use agreement requirements.

## Supplementary Materials

The following supporting information can be downloaded at: https://www.mdpi.com/article/10.3390/cancers18172795/s1. Table S1: Individual intervals between tumor resection and the TP1 fMRI examination. Table S2: Sensitivity analyses of the nested cross-validation classification pipeline. In the speech-history analysis, the eight participants with postoperative complete mutism (n = 3) or reduced speech (n = 5) were excluded before any feature selection or model fitting, and the pipeline was rerun from scratch in the remaining 23 participants. In the motion-adjusted analysis, first-level GLMs were re-estimated with the six rigid-body realignment parameters (three translations and three rotations) included as nuisance regressors for each functional run. The 95% confidence intervals are exact Clopper–Pearson intervals; permutation p values were obtained using the same one-sided within-subject 20,000-label-swap procedure as in the primary analysis. MAD, median absolute deviation.
