# Supplementary material for: Silent Verb Generation During fMRI Reveals Post-Radiotherapy Alterations in Cerebellar–Cerebral Language Regions in Patients Treated for Medulloblastoma
Source: Cancers (Basel). 2026 Aug 28;18(17):2795. doi: 10.3390/cancers18172795 (PMC13564590; doi:10.3390/cancers18172795)
Supplement: Supplementary file 1 [file cancers-18-02795-s001.zip › cancers-4348840-supplementary.pdf]

Supplemental Material

Table S1. Individual intervals between tumor resection and the TP1 fMRI examination.

Patient	Interval (days)
1	34
2	36
3	24
4	22
5	22
6	24
7	28
8	15
9	32
10	33
11	31
12	28
13	33
14	40
15	35
16	34
17	15
18	23
19	33
20	35
21	27
22	23
23	42
24	27
25	44
26	28
27	13
28	34
29	30
30	26
31	23

Table S2. Sensitivity analyses of the nested cross-validation classification pipeline. In the speech-history analysis, the eight participants with postoperative complete mutism (n = 3) or reduced speech (n = 5) were excluded before any feature selection or model fitting, and the pipeline was rerun from scratch in the remaining 23 participants. In the motion-adjusted analysis, first-level GLMs were re-estimated with the six rigid-body realignment parameters (three translations and three rotations) included as nuisance regressors for each functional run. The 95% confidence intervals are exact Clopper–Pearson intervals; permutation p values were obtained using the same one-sided within-subject 20,000-label-swap procedure as in the primary analysis. MAD, median absolute deviation.

Analysis	n	Accuracy	95% CI	Permutation p
Primary fully nested pipeline (reference)	31	22/31 (71.0%)	52.0–85.8%	0.015
Exclude postoperative complete mutism/reduced speech	23	16/23 (69.6%)	47.1–86.8%	0.049
Median/MAD sphere ranking	31	21/31 (67.7%)	48.6–83.3%	0.035
Rank-based sphere ranking	31	20/31 (64.5%)	45.4–80.8%	0.075
3-voxel searchlight radius	31	19/31 (61.3%)	42.2–78.2%	0.140
Motion-adjusted first-level GLMs	31	22/31 (71.0%)	52.0–85.8%	0.017
